# *Drosophila* eIF4E paralogs: a paradigm of functional divergence

**DOI:** 10.1080/15476286.2026.2753988

**Published:** 2026-10-28

**Authors:** Greco Hernández, Daniela Ross-Kaschitza, Julie A. Brill, Brook L. Falk, Paul Lasko, Nahum Sonenberg

**Affiliations:** a MRNA and Cancer Laboratory, Subdirección de Investigación Básica, Instituto Nacional de Cancerología (INCan; National Institute of Cancer), Mexico City, Mexico; b Institut für Biochemie und Molekulare Medizin (IBMM), University of Bern, Bern, Switzerland; c Department of Molecular Genetics, University of Toronto, Toronto, ON, Canada; d Cell & Systems Biology Program, Sickkids Research Institute, Toronto, ON, Canada; e Department of Biology, McGill University, Montreal, QC, Canada; f Department of Biochemistry and Goodman Cancer Institute, McGill University, Montreal, Canada

**Keywords:** Translation initiation, eIF4E, translational control, drosophila, development

## Abstract

A cardinal factor in the regulation of mRNA life and function is the cap-binding protein eIF4E. eIF4E promotes the recruitment of mRNA to the ribosome during the initiation step of translation, but other activities have been described, including mRNA processing, export, and storage. To perform these functions, eIF4E must associate with the mRNA cap and a number of different eIF4E-interacting proteins (4E-IPs). Thus, eIF4E has evolved as a key interface between RNA metabolism, gene expression, and development. In *Drosophila melanogaster,* seven genes encode eight eIF4E cognate proteins that have functionally diverged to perform different activities. We review the structure, interactors, biochemistry, and activities of the *Drosophila* eIF4E paralogs, which represent a paradigm of divergence during metazoan development.

## Introduction

Eukaryotic translation initiation factor (eIF) 4E binds the cap structure at the 5´-end of mRNAs and interacts with many partner proteins [1,2]. These properties make eIF4E a versatile protein involved in different processes of mRNA metabolism, such as translation, nuclear export, and storage in cytoplasmic foci [3]. During eukaryotic evolution, *eIF4E* underwent successive duplications in different lineages, and the cognate genes acquired specialized functions in specific tissues or processes. In the fly *Drosophila* genus, species possess 1–7 cognate *eIF4E* genes encoding proteins that originated by multiplication during the explosive evolutionary radiation of this lineage in the early Tertiary [4]. Thus, genes *eIF4E-1,2*, *eIF4E-3*, *eIF4E-4*, and *eIF4E-5* cluster together in the distal region of the left arm of chromosome 3; genes *eIF4E-6*, and *4E-HP* (also termed *eIF4E-8*) lie in the distal region of the right arm of chromosome 3; and *eIF4E-7* lies on chromosome X. Figure 1 shows the chromosomal location of eIF4E paralogs.

**Figure 1.**
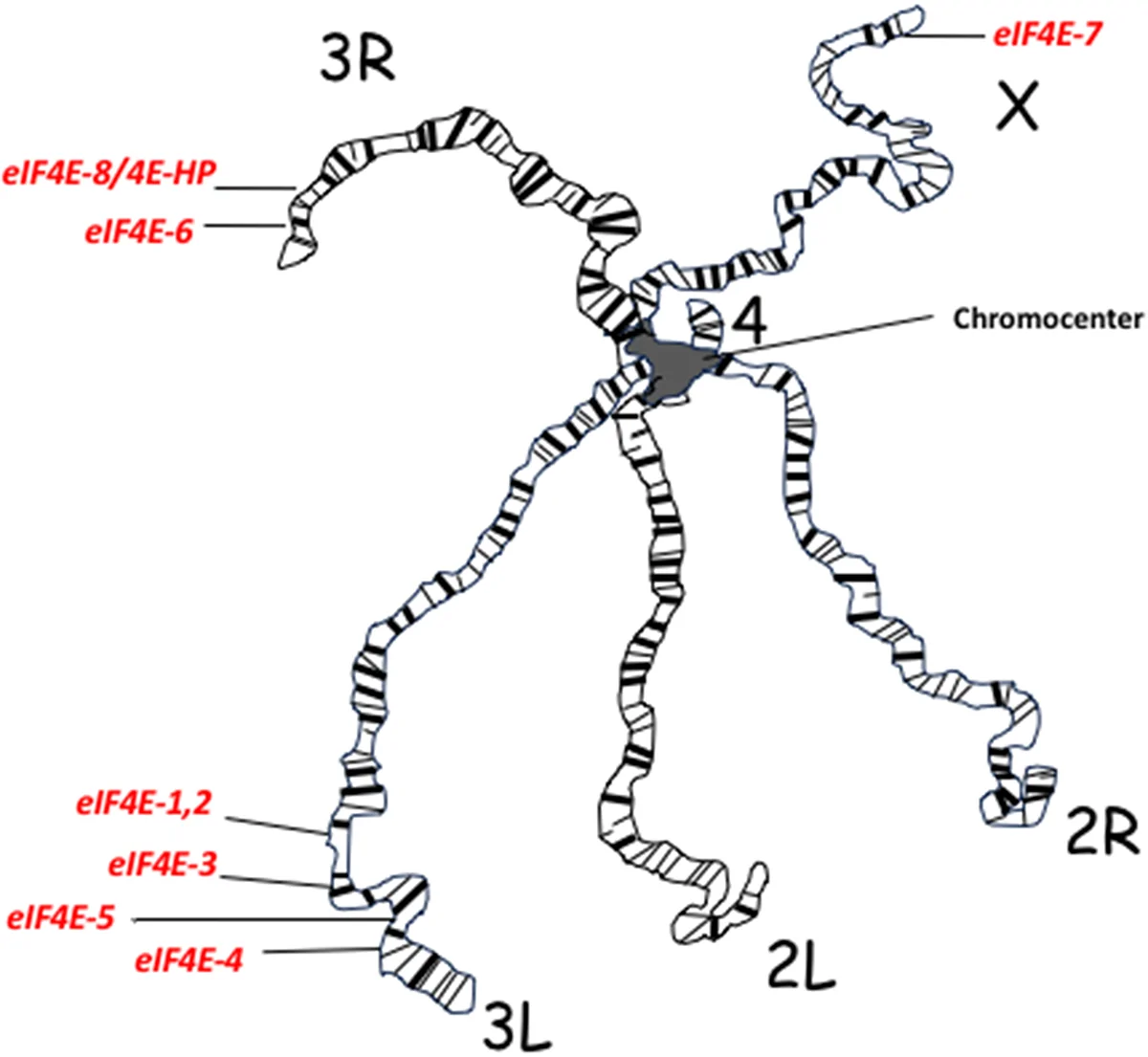
Cytogenetic map of the eIF4E paralog genes. A cartoon is depicted representing polytene chromosomes and the cytogenetic location of *D. melanogaster* eIF4E genes. Only some bands are shown. *eIf4E-1,2* (gene id *CG4035*): 3 L, division 67B4; *eIf4E-3* (gene id *CG8023*): 3 L, division 66C1; *eIf4E-4* (gene id *CG10124*): 3 L, division 65C1; *eIf4E-5* (gene id *CG8277*): 3 L, division 66A18; *eIf4E-6* (gene id *CG1442*): 3 R, division 98F5; *eIf4E-7* (gene id *CG32859*): X, division 1E3; *eIf4E-8/4E-HP* (gene id *CG33100*): 3 R, division 95D11-95E1. For sequence location, see FlyBase.Org. *X*, chromosome X; *2 L*, left arm of chromosome 2; *2 R*, right arm of chromosome 2; *3 L*, left arm of chromosome 3; *3 R*, right arm of chromosome 3; *4*, chromosome 4. Modified from [5,6].

*D. melanogaster* is a model organism, key in the study of development, which contains seven cognate genes encoding eight eIF4E paralogs. eIF4E from all organisms is classified according to the nature of the amino acids that are involved in cap-binding, namely W56, W102, E103, R/K112, R/K157, K/R162, and W166 (human protein numbering). Class I proteins contain all these conserved residues, and Class II proteins contain the residues W102, E103, R/K112, R/K157, and W166, but have the conservative change W56 ➜ Y/F and the non-conservative substitutions K/R162 ➜ I/V/L [7]. *Drosophila* eIF4E-1 to −7 are Class I proteins, whereas 4E-HP belongs to Class II. Among them, eIF4E-1 and 4E-HP/eIF4E-8 are the best characterized. Throughout the life cycle of *Drosophila*, eIF4Es are essential for different processes in distinct tissues. In this review, we describe the functional divergence that eIF4E paralogs have undergone in *Drosophila*.

## Biochemical function of eIF4Es

The primary biochemical function of eIF4E paralogs is to bind to the 5’-end m^7^G cap of mRNAs [8]. Moreover, the specific biological activity in which each paralog is involved (i.e. mRNA translation promotion or inhibition, nucleo-cytoplasmic transport or storage) is determined by the type of interactors and alternative complexes that they can form in the cell [3]. Indeed, *Drosophila* eIF4Es differentially bind to a plethora of eIF4E-interacting proteins (Table 1). However, some of the reported interactions have been tested only by one methodology, for instance, yeast two-hybrid or co-immunoprecipitation, and the described genetic interactions may not reflect direct biochemical functions. Thus, further experiments using alternative methods should be performed to corroborate these interactions *in vivo*.

**Table 1.** IInteractors^a^ of *Drosophila* eIF4Es.

Protein^b,c^	eIF4G	eIF4G-2	4E-BP	Mextli	SERCA	CUP	4E-T	Me31B	Bicoid	Belle	Ref.
eIF4E-1	+ (2 H; Co-IP; PD; FBA; ITC; 3D)	+ (2 H; Co-IP)	+ (2 H; Co-IP; ITC; 3D)	+ (2 H; Co-IP; PD; 3D)	+ (2 H)	+ (Co-IP; PD; ITC; DFS; 3D)	+ (Co-IP; PD; ITC; 3D)	+ (2 H; Co-IP; FRET)	+ (PD, Co-IP)	+ (Co-IP)	[9–27]
eIF4E-2	+ (2 H; FBA)	NT	+ (2 H)	+ (2 H)	+ (2 H)	NT	NT	NT	NT	NT	[10–12,19,26]
eIF4E-3	+ (2 H)	+ (2 H; Co-IP)	-(2 H; Co-IP)	+	NT	NT	NT	+ (2 H Co-IP; FRET)	NT	NT	[10,12,19,20]
eIF4E-4	+ (2 H; FBA)	NT	+ (2 H)	+	+ (2 h)	NT	NT	NT	NT	NT	[10–12,19,26]
eIF4E-5	+ (2 H; FBA)	+ (2 H)	+ (2 H)	NT	NT	+ (2 H)	+ (2 H)	NT	NT	NT	[10–12,28]
eIF4E-6	–	NT	-(2 H)	-(2 H)	-(2 H)	NT	NT	NT	NT	NT	[10,19]
eIF4E-7	+ (2 H; FBA)	+ (2 H)	+ (2 H)	+ (2 H)	-(2 H)	NT	NT	NT	NT	NT	[10–12,26]
4E-HP	-(2 H; FBA)	NT	-(2 H)	-(2 H)	-(2 H)	NT	NT	NT	+	+ (Co-IP;PD)	[10–12,21,26,29]

^a^Assay used to prove direct interaction: *2 H*, yeast two-hybrid; *co-IP*, co-immunoprecipitation; *PD*, pulldown; *FBA*, fluorescence binding assay using the eIF4G peptide 618-KKQYDREQLLQLR-630; *ITC*, isothermal titration calorimetry; *FRET*, Fluorescence resonance energy transfer; *3D*, crystal structure; *DSF*, Differential scanning fluorimetry; *NT*, non-tested.

^b^*eIF4G*, gene *CG10811*; *eIF4G2*, gene *CG10192*; *4E-BP* (*Thor*), gene *CG8846*; *Mextli*, gene *CG2950*; *SERCA*, gene *CG3725*; *CUP*, gene *CG11181*; *4E-T*, gene *CG32016*; *Bicoid*, gene *CG1034*.

^c^Caveat: Co-IP experiments cannot establish direct interaction between two proteins, as it is always possible that any detected interactions could be mediated by one or more other proteins. The same goes for pulldown experiments, unless the proteins tested have been purified from bacteria.

eIF4E-1 was first characterized [9,30] and cloned [31] from embryos. Subsequently, it was discovered that the gene *eIF4E-1,2* expresses three mRNAs produced by alternative splicing, which encode eIF4E-1 and eIF4E-2 [32,33]. *eIF4E-1,2* alternative splicing is mediated by Half-pint, a regulator of RNA processing [34,35]. Subsequently, the genes *eIF4E-3*, *-4*, *-5*, *-6*, *-7*, and *4E-HP* (*eIF4E-8*) were identified *in silico* [36], and biochemically [10–13] and structurally [14–16] characterized. All *D. melanogaster* eIF4E proteins bind to cap analogs, albeit with a 14-fold affinity difference among them [11] (Table 2). This wide range of affinities may allow eIF4E paralogs to promote different magnitudes of translation initiation.

**Table 2.** AlphaFold3 predicted distances (Å units) between the m^7^GpppG cap-interacting amino acids and the cap moieties of *Drosophila* eIF4Es^a^, and K_as_ with the cap.

Cap-binding amino acid	W/Y	W	E	W	R/Q	R/K	K/S	Equilibrium association constant (K_as_) with m^7^GpppG (μM^−1^)^b^
Cap moiety	Pi-Pi stacking	Pi-Pi stacking	Guanine ring	7-methyl	Phosphate	Phosphate	Phosphate	
eIF4E-1	3.7	3.9	2.8	3.0	4.6	3.4	3.7	1.29 ± 0.04
eIF4E-2	3.9	3.7	2.5	3.7	4.6	3.6	3.7	1.23 ± 0.04
eIF4E-3	4.2	4.0	2.8	4.3	4.6	2.7	3.3	8.24 ± 0.28
eIF4E-4	3.7	3.7	2.7	4.2	5.3	2.6	4.1	0.38 ± 0.01
eIF4E-5	2.4	3.3	2.3	3.8	4.5	3.4	3.1	4.44 ± 0.2
eIF4E-6	4.0	4.1	3.5	3.9	5.2	2.7	4.3	Non -determined
eIF4E-7	4.2	3.6	2.7	3.9	4.6	3.3	3.5	1.56 ± 0.1
4E-HP	4.1	2.9	2.7	3.9	6.6	1.6	5.7	0.84 ± 0.03

^a^Data from the cap-binding pocket shown in Figure 2.

^b^Taken from Zuberek *et al*. 2016 [11].

**Figure 2.**
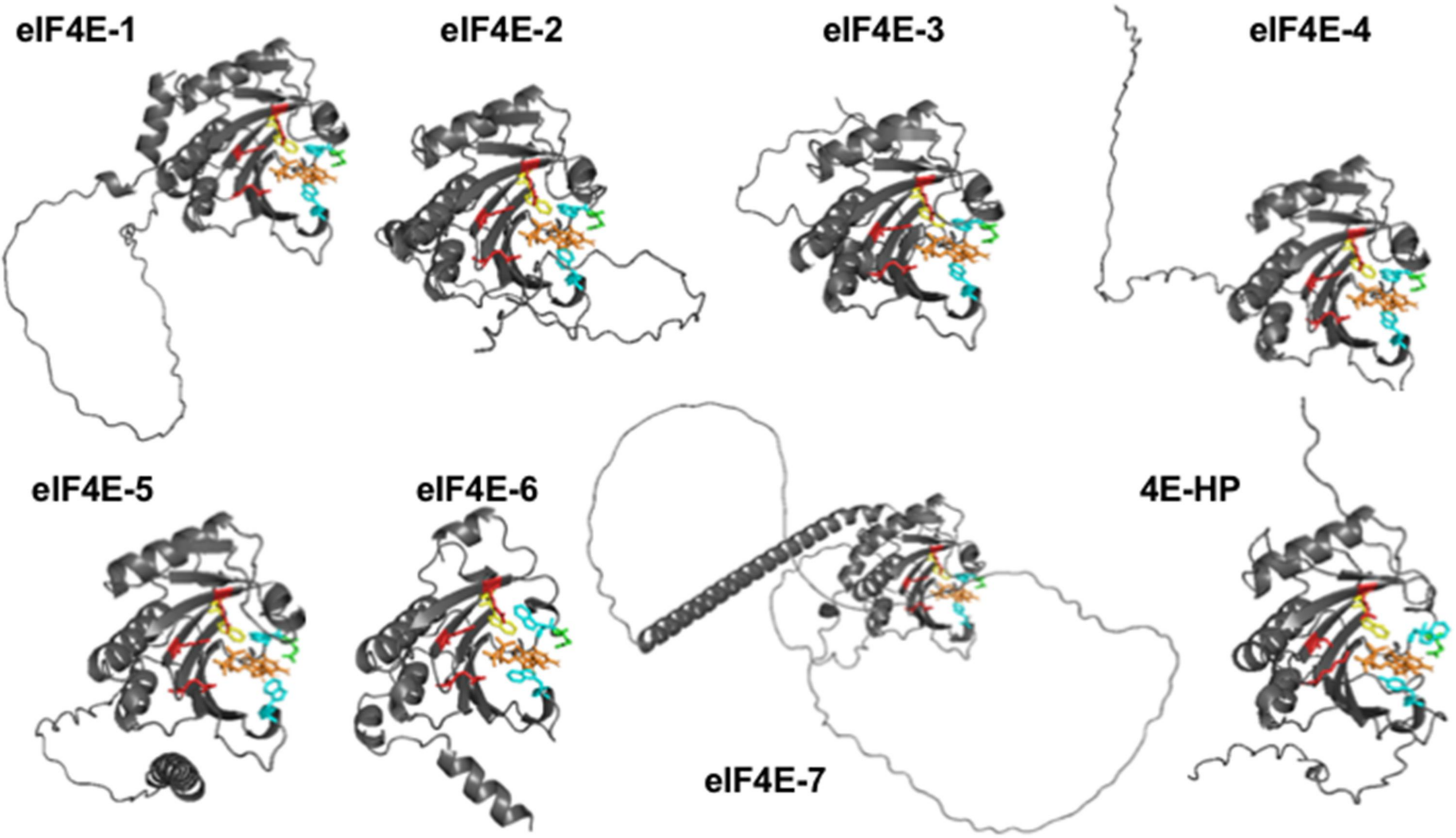
Global three-dimensional structure predictions of *D. melanogaster* eIF4Es. The indicated proteins (*gray*) and the m^7^GpppG cap (*orange*) are depicted. The amino acids involved in cap recognition are colored as in Figure 2. The structures were predicted using AlphaFold3 and aligned with the eIF4E‑1 3D structure (PDB id: 4UEC).

The first and best‑characterized activity of eIF4E is the promotion of mRNA translation in mammals [1,2]. This is its predominant role in all cells. In this process, eIF4E binds the scaffold eIF4G protein and the RNA helicase eIF4A to form the eIF4F complex and recruit an mRNA via cap-binding. eIF4G then recruits the 40S ribosomal subunit by interacting with ribosome-attached eIF3 [2]. In mammals, two signaling cascades control translation initiation by targeting eIF4E, which either down- or upregulate mRNA cap-binding by eIF4F. These are the phosphatidylinositol 3-kinase (PI3K)/protein kinase B (Akt)/mammalian target of rapamycin complex 1 (mTORC1) pathway and the mitogen-activated protein kinases (MAPKs) pathway [37,38]. The mTORC1 pathway targets eIF4E via eIF4E-binding proteins (4E-BPs 1, 2, and 3), which bind and inhibit eIF4E. Hypophosphorylated 4E-BPs sequester eIF4E, blocking formation of the eIF4E/eIF4G complex and translation initiation, whereas phosphorylated 4E-BPs dissociate from eIF4E, allowing eIF4E/eIF4G binding and promoting translation [39]. This occurs in response to energy status, hormones, mitogens and nutrients. In response to stress and mitogens, the MAPK cascade activates mitogen-activated protein kinase interacting kinase 1 and 2 (MNK1/2) that phosphorylates eIF4E at Ser209 (human protein numbering). Phosphorylated eIF4E promotes selective translation of a subset of mRNAs [37,38].

These pathways are highly conserved in *Drosophila*. *Drosophila* 4E-BP (also called Thor) is 38% identical and 67.5% similar to human 4E-BP2 [40]. Thor negatively regulates eIF4E-1 downstream of the PI3K/Akt/TOR pathway to control cellular and organismal growth in response to environmental, endocrinological, and nutritional cues [40–44]. Unlike eIF4E-1, eIF4E-3, eIF4E-6, and 4E-HP do not bind 4E-BP [10,12]. Whether other *Drosophila* eIF4Es are regulated by the PI3K/Akt/TOR cascade is unknown. eIF4E-1 phosphorylation at Ser251 (equivalent to human Ser209) is critical for *Drosophila* development [45]. Transgenic animals expressing a non-phosphorylatable Ser251Ala eIF4E-1 mutant in an *eIF4E-1,2* mutant background showed reduced viability, indicating that Ser251 phosphorylation is essential for normal growth and development [45]. This contrasts with mutant mice carrying a non-phosphorylatable form of eIF4E, in which only the translation of a subset of mRNAs is affected [46]. In human cells, phosphorylation of eIF4E promotes translation also of selective mRNAs [47,48].

It was subsequently demonstrated that kinase LK6 is the *Drosophila* ortholog of mammalian MNK kinases that phosphorylates eIF4E-1 downstream of MAPK [49–51]. Indeed, *lk6* mutants exhibit decreased viability, delayed development, and smaller adult size, demonstrating that LK6 activity is required for organismal growth and development [49–51]. Although eIF4E-2, −5, −7, and 4E-HP possess the conserved Ser251, there are no reports that these paralogs are phosphorylated downstream of the MAPK cascade.

## Structural features of eIF4E regulation

Three-dimensional studies revealed that mammalian and yeast eIF4Es are spherical, cupped-hand shaped proteins containing a cavity formed by a curved eight antiparallel beta-strands overlaid by three α-helices. W56 and W102 (human protein numbering) within the protein pocket interact with the cap via sandwich-like Pi-stacking interactions and a van-der-Waals bond of W166 with the cap methyl group. The positively charged residues R112, R157, and K162 form additional salt bridge interactions with the ribose and triphosphate moieties of the cap. Association of E103 with the guanine ring further stabilizes cap binding [52–55].

In *Drosophila*, 3D crystal structures of full-length or fragments of eIF4E-1 in complex with Cup, 4E-BP, regions of Mextli, eIF4G, and 4E-transporter (4E-T) were elucidated [14–17]. So far, no other *Drosophila* paralog has been studied at the 3D level. The N-terminal 79 amino acids of eIF4E-1 are unstructured, and amino acids 80–248 form a core with the same 3D structure as the mammalian and yeast orthologs. AlphaFold three-dimensional structural predictions of the other *Drosophila* eIF4Es, based on the known structure of eIF4E-1 [14–17], suggest the cupped-hand shape is a common characteristic of *Drosophila* eIF4Es (Figure 2). The N-termini of eIF4Es vary in length and are predicted to be unstructured. eIF4E-7 possesses the longest N-terminal sequence (249 amino acids). We performed molecular modeling to predict cap interactions within the cap-binding pocket and observed variations in the spatial arrangement of the amino acids contacting the cap (Figure 3). The predicted distances between the cap and the cap-binding amino acids were determined (Table 2). For comparison, we included the experimentally observed equilibrium association constant (K_as_) with the m^7^GpppG cap measured using recombinant proteins, as determined by Zuberek *et al*. [11]. eIF4E-3 and eIF4E-4 show the highest and the lowest cap-binding affinities, respectively [11].

**Figure 3.**
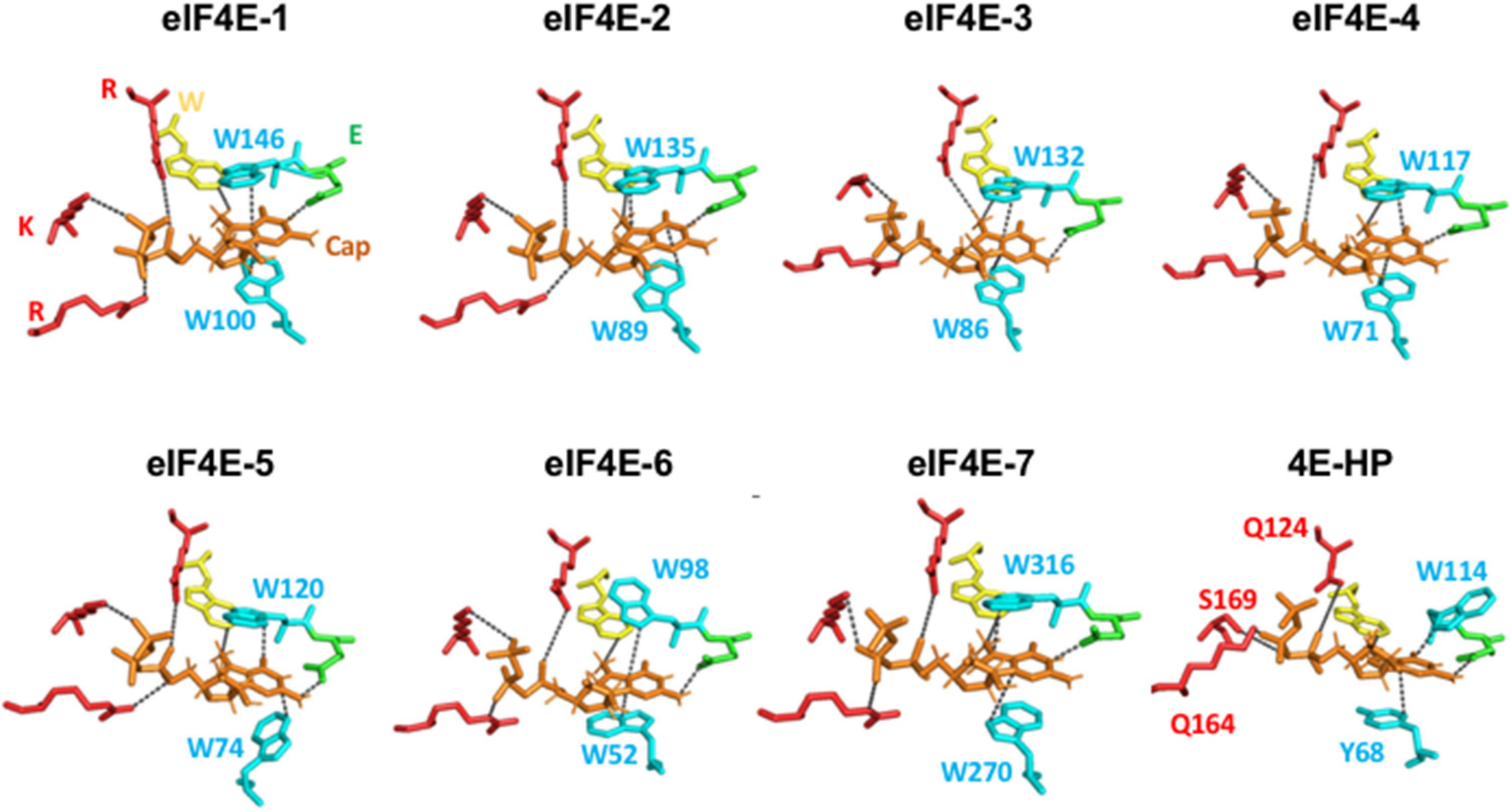
Three-dimensional structure prediction of the cap-binding pocket. Detailed views of the *Drosophila* eIF4Es in complex with the m^7^GpppG cap (*orange*) are depicted. Amino acids involved in cap recognition of the indicated eIF4Es are shown. Aromatic residues interacting via sandwich-like Pi▯-stacking bonds with the cap methyl group are indicated for all proteins in *blue*. Positively charged R, R, and K residues forming additional salt bridge interactions with the ribose and triphosphate moieties of the cap are shown in *red*. The E residue that associates with the guanine ring of the cap is shown in *green*. Only 4E-HP possesses the indicated substitutions. Structures were predicted with AlphaFold3 and aligned with the eIF4E‑1 3D structure.

The eIF4E partners 4E-BP, Cup, and 4E-T possess a bipartite binding site composed of a canonical eIF4E-binding motif (4E-BM) YXXXXL▯ (where X is any amino acid and ▯ is any hydrophobic residue) [56,57] and a second, non-canonical region not conserved at the sequence level that binds to a hydrophobic cavity on the lateral surface of eIF4E about 30 residues away from the canonical motif. The non-canonical 4E-BM is lined by a conserved isoleucine and tyrosine (I122 and Y119 in eIF4E-1). The region between the two binding motifs forms an elbow loop that bends the protein backbone 90 degrees downward [13–15,17]. In contrast, eIF4G possesses only the canonical binding motif [13,15,18]. Thus, 4E-BP, Cup and 4E-T dock onto the eIF4E–eIF4G complex and displace eIF4G to repress translation [13,58]. The bipartite interaction of different eIF4E‑interacting proteins (4E-IPs) occurs in fungi and mammals and appears to be conserved across eukaryotes [13–17,58,59]. A sequence comparison of the structured and conserved eIF4E core showed that the residues H81–M84 and N105–P125 of eIF4E-1, which contact the canonical and non-canonical 4E-BM of partner proteins, respectively, are highly conserved in the other eIF4E paralogs (Figure 4), except for 4E-HP and eIF4E-6, which do not bind eIF4G and 4E-BP [10]. AlphaFold3 analysis of 3D structure for both regions predicted that Arg34 and Arg47 of eIF4E-6 and 4E-HP, respectively, distort the region binding the canonical 4E-BM (Figure 4(B)). Moreover, a shorter alpha-helix (for eIF4E-6) and a longer one (for 4E-HP) are predicted for the region contacting the non-canonical 4E-BM, in comparison with the rest of eIF4Es (Figure 4(C)). These regions of eIF4E-6 and 4E-HP could be involved in unidentified, novel partner proteins.

**Figure 4.**
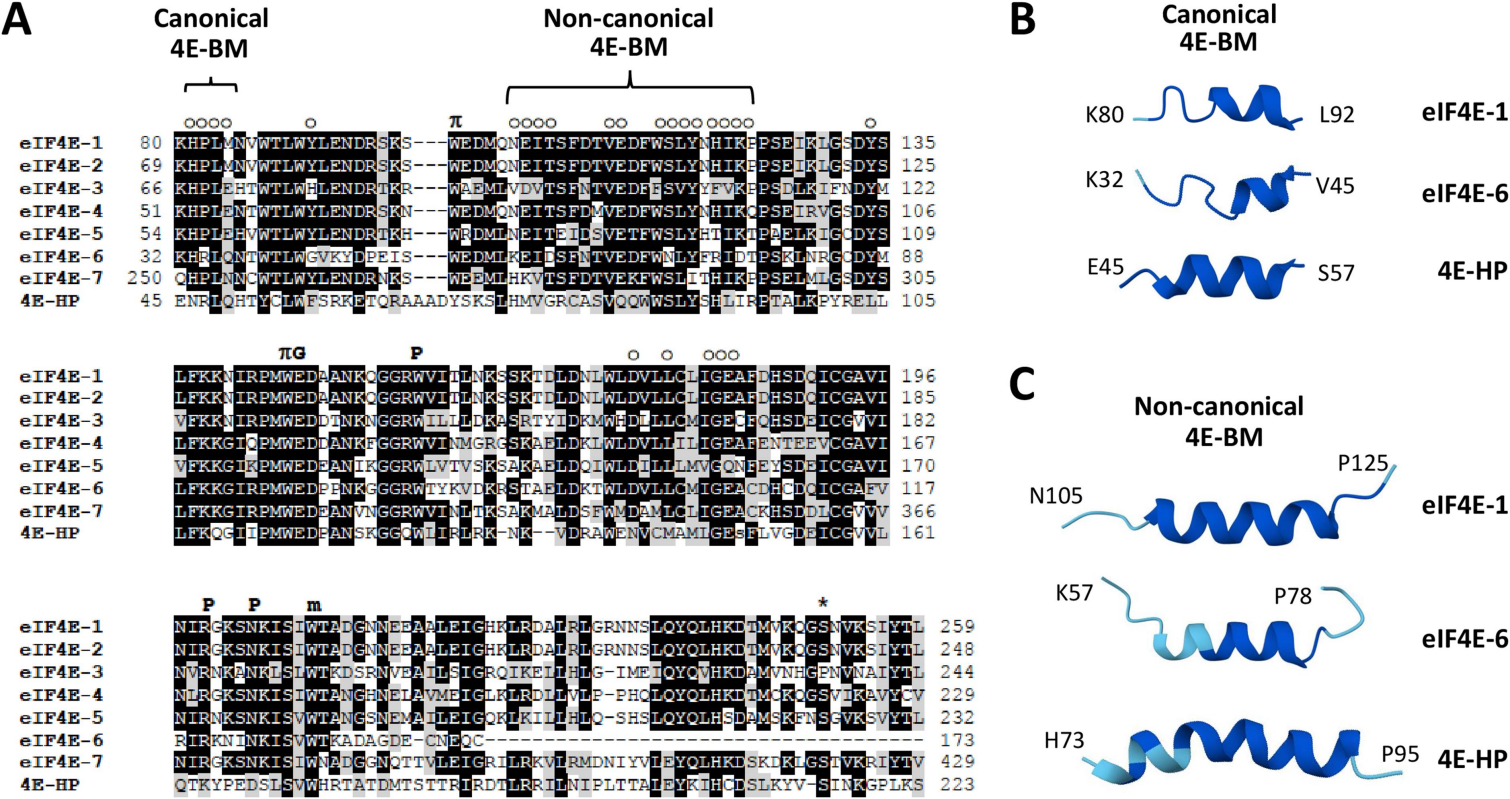
Sequence and predicted structure comparison of *D. melanogaster* eIF4E paralogs. *A)* sequence comparison of the proteins’ core region. Residues identical in at least five proteins are shaded in black boxes. Conservative residues in at least five sequences are in gray boxes. Residues involved in cap recognition [52–54,60] are indicated as follows: Pi, W binding the guanine by Pi-Pi interactions; *G*, residue recognizing the guanine ring; *P*, residues interacting with the phosphate groups; *m*, W recognizing the cap methyl group. Residues of eIF4E‑1 contacting the canonical eIF4E-binding motif (4E-BM) (YXXXXLfi) or the non-canonical 4E-BM of eIF4G, 4E-BP, 4E-T, Mextli, or cup [13–17] are indicated with circles. The phosphorylatable S251 of eIF4E‑1 critical for development [45] is marked with an asterisk. Sequences were obtained from FlyBase (http://flybase.Org) and aligned using the clustal omega program (https://www.Ebi.ac.uk/tools/msa/clustalo/). The following conservative amino acids were considered: G and A; S and T; K, R and H; E, D, Q and N; and L, I, M, V, C, Y, F, and W. *B)* and *C)* AlphaFold3 [61] 3D structure prediction of selected proteins. *B)* sequence encompassing the residues that contact the canonical 4E-BM of eIF4E-interacting proteins. *C)* region contacting the non-canonical 4E-BM of eIF4E-interacting proteins.

In other eukaryotes, such as the unicellular and the parasitic Trypanosomatids, insertions and N- and C-terminal extensions in some of the eIF4E paralogs are determinants for protein–protein interactions and alternative protein complex formation [62]. Indeed, the highly variable amino-terminal region of *Drosophila* eIF4E paralogs could bind a variety of unknown binding proteins to form complexes with specific functions in different tissues, worth investigating. In contrast, the *Drosophila* proteins (except eIF4E-6) possess a highly conserved core region with a very similar size (equivalent to residues 80–259 of eIF4E-1; Figure 4(A)).

eIF4E-binding domains show diverse architecture in *Drosophila*. For example, Mextli is a 70-kDa *Drosophila* protein that binds multiple eIF4E paralogs and eIF3 to promote translation during oogenesis. Thus, Mextli represents a functional eIF4G analog [19]. Mextli evolved an unorthodox, tripartite eIF4E‑binding mode in which the bipartite binding region is extended by an additional auxiliary linker and a third 4E-BM that bends back and associates with the dorsal surface of eIF4E antiparallel to the canonical motif [16]. It would be worth investigating whether and how other *Drosophila* eIF4Es bind the different 4E-IPs (Table 1).

## eIF4E-1 supports global translation during development

eIF4E-1 binds to eIF4G or fragments of it via a canonical 4E-BM [9–11,13,15–18,63] and promotes translation *in vitro* [9,30,64] and *in vivo* in a rescue assay of a yeast strain lacking endogenous eIF4E [10]. Functional [9,30] and proteomic [10] analyses of the embryonic cap-binding complex identified eIF4E-1 as the sole protein binding to the cap. Accordingly, it was observed that different *eIF4E-1,2* mutant alleles arrest growth during early larval development, and that some showed embryonic lethality and widespread apoptosis [10,45,65]. Altogether, these observations demonstrated that the bulk of translation during embryogenesis relies solely on eIF4E-1.

eIF4E-1 is detected ubiquitously throughout the complete life cycle across tissues, where it might support routine translation [12] (Figure 5(A)). In addition to embryogenesis and early larva, the role of eIF4E-1 has been addressed in some adult tissues as well. In the developing adult eye, which is composed of cells forming a lattice of a precise number of ommatidia and chaetae (bristles), eIF4E-1 loss-of-function mutations inhibited cell growth but did not affect differentiation [66]. In contrast, ectopic overexpression of eIF4E-1 in the eye imaginal disc leads to cell proliferation in the ommatidia and the presence of extra chaetae in the adult eye [10]. These observations support the notion that eIF4E-1 supports translation and cell growth in the eye. The role of eIF4E-1 and other paralogs in spermatogenesis is discussed later.

**Figure 5.**
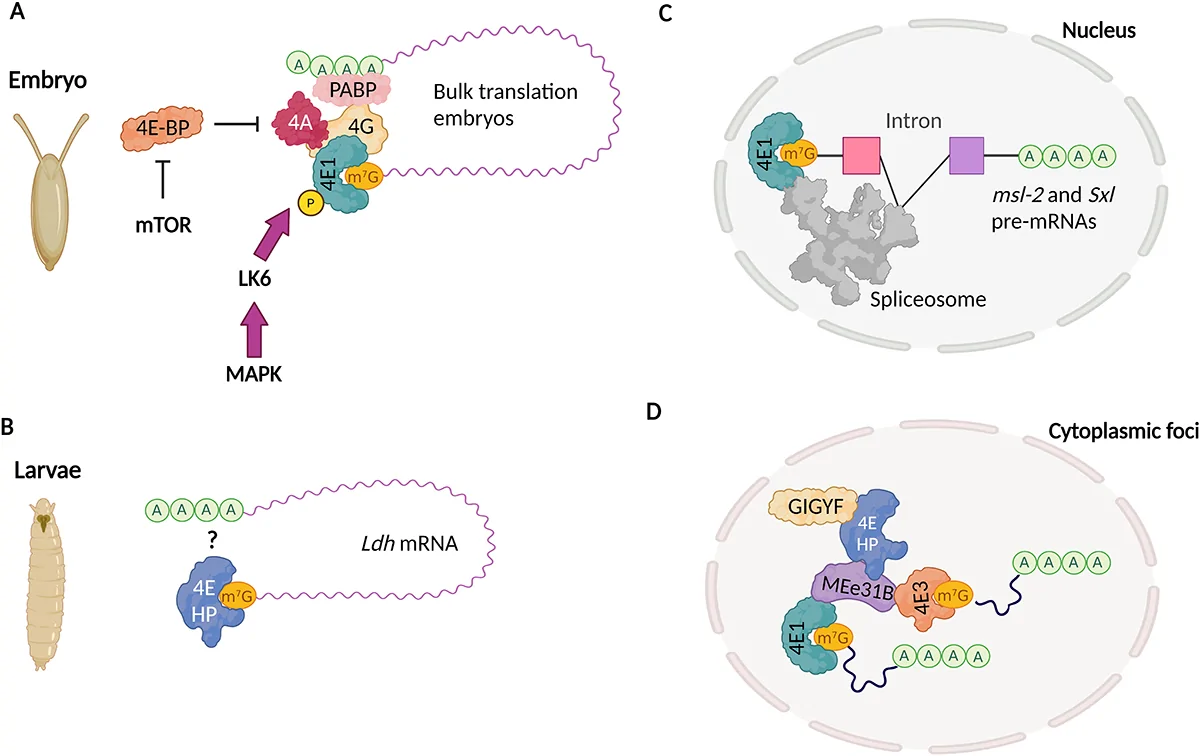
Diverse roles of *D. melanogaster* eIF4E‑1 and 4E-HP. *A)* canonical and global translation is performed by eIF4E‑1 (*4E-1*). Formation of the eIF4E‑1/eIF4G complex is regulated by the mTOR/4E-BP axis. eIF4E‑1 is phosphorylated by MAPK/Lk6 kinases. *B)* translation of the hypoxia-responsive *Ldh* mRNA is promoted by 4E-HP (*HP*). *C)* during sex determination, eIF4E‑1 associates with the spliceosome and promotes splicing of *msl-2* and *Sxl* pre-mRNAs in the nucleus. *D)* eIF4E‑1 and eIF4E‑3 (*4E-3*) associate with the RNA helicase Me31B to store mRNAs within cytoplasmic foci. 4E-HP associates with GIGYF, and perhaps Me31B, in these membrane-less structures. Image created using BioRender.com.

## 4E-HP is needed for translational responses to stress

In eukaryotes, translation is severely downregulated under different stresses. 4E-HP is involved in stress responses. *Drosophila* is highly resistant to an anaerobic environment. A key component of the hypoxia response is Lactate dehydrogenase (Ldh), whose mRNA is highly translated under this condition by a mechanism involving a CA-rich motif present in its 3´-untranslated region (3´-UTR). Recently, it was reported that 4E-HP promotes translation of *Ldh* mRNA under anaerobic conditions and that 4E-HP is necessary for *Drosophila* development [67] (Figure 5(B)).

In healthy cells, different networks operate to ensure the maintenance of protein homeostasis also termed proteostasis. During aging, numerous external and internal stresses accumulate that lead to the loss of proteostasis, which leads to the accumulation of misfolded protein aggregates and, ultimately, destabilizes the proteome integrity [68]. The stress-induced Activating Transcription Factor 4 (ATF4) is a major transcription factor that mediates the adaptive mechanism to proteostasis stress. In *Drosophila*, ATF4 (encoded by *cryptocephal*) translation is regulated via upstream open reading frames (uORFs) in the *ATF4* 5´-untranslated region (5´-UTR), like in vertebrates [69]. Recently, it was reported that 4E-HP, along with NELF-E, the 40S ribosome subunit, and eIF3, comprise an ATF4 regulatory network required for the ATF4 response to dysfunctional proteostasis stress in the larval fat body [70]. Whether 4E-HP directly promotes the translation of ATF4 mRNA is not known.

## Involvement of eIF4E-1 in nuclear pre-mRNA splicing

eIF4E is a cytoplasmic translational factor in all studied species. In monkey, human, and mouse cells, eIF4E has also been found inside the nucleus, where it mediates mRNA export or 3´‑end pre-mRNA processing [71–80]. In other species, including *Trypanosoma brucei* [81], *Giardia* [82], *Xenopus* [83], and *S. cerevisiae* [84,85], eIF4E orthologs have also been detected in the nucleus, but their biological role in this organelle in these species is unknown.

In *Drosophila* embryos [86] and cultured S2 cells [13], eIF4E-1 also has been found within the nucleus. An embryonic genetic screen revealed that *eIF4E-1,2* genetically interacts with *Sex-lethal* (*Sxl*) to repress expression of the male-specific lethal-2 (Msl-2) protein that is involved in fly sex determination [77]. Surprisingly, eIF4E-1 is not required for repression of *msl-2* mRNA translation. Instead, eIF4E-1 binds *Sxl* pre-mRNAs as well as several splicing factors during the assembly of spliceosome complexes (Figure 5(C)) and acts as a co-factor in the female-specific splicing of *msl-2* and *Sxl* mRNAs.

The presence of nuclear eIF4E in multiple species strongly suggests that this may be a universal feature across eukaryotes. In *Drosophila*, however, no paralog other than eIF4E-1 has been detected in the nucleus. Nevertheless, it is worth searching for nuclear localization of more eIF4Es in this organism. Given that eIF4E activity and subcellular localization depend on its interactions with specific binding partners [3,80], the identification of novel nuclear 4E-IPs in *Drosophila* will provide new insights into the biological functions of nuclear eIF4E.

## mRNA storage and decay in cytoplasmic foci

In eukaryotic cells, translational control, storage and degradation of mRNAs occur in different cytoplasmic ribonucleoprotein foci, which include the processing bodies (PB) and stress granules (SG) [87,88]. When mRNAs are not translated, they can be transiently stored or even degraded in these foci. In addition to the role in translation in the cytoplasm and its roles within the nucleus, eIF4E can also play a role in mRNA storage and turnover in the cytoplasmic foci. For silent mRNAs, localization to PB and SG depends on ribonucleoprotein (RNP) complexes containing numerous proteins and transcripts. In *Drosophila*, non-translated mRNAs are stored in PB, where the RNA helicase Me31B (DDX6 in vertebrates) assembles complexes with the silent mRNAs and the factors Exuperantia, Cup, and eIF4E-1 to cause translational repression [89–93] (Figure 5(D)). Indeed, Me31B interacts with eIF4E-1 and eIF4E-3, and colocalizes with 4E-HP in cultured S2 cells [20,94]. Accordingly, Ruscica *et al.* [95] reported that 4E-HP makes a complex with GRB10-interacting GYF (glycine-tyrosine-phenylalanine domain) protein (GIGYF) to couple repression of mRNA translation with mRNA stability in S2 cells. In this process, GIGYF recruits Me31B, the decapping activator HPat, and the CCR4-NOT deadenylase complex to repress translation and to promote mRNA decay.

Although the role of these eIF4E paralogs in cytoplasmic foci has been well characterized in cultured S2 cells, further investigation should be done in different tissues and throughout the *Drosophila* development to better understand the translational repression, storage and degradation of mRNAs *in vivo*.

## The critical roles of eIF4Es in development

During embryogenesis, embryo axes and body patterning are established by the action of several morphogens. The embryo further gives rise to three consecutive instar larvae, a pupal stage, and adulthood [96]. *Drosophila eIF4E* genes are differentially expressed throughout development, with the mRNAs showing differential spatial distribution. *eIF4E-1,2* mRNAs strongly accumulate in pole cells at the posterior end of the embryo that gives rise to the germline. Subsequently, the *eIF4E-1,2* mRNAs are highly expressed throughout the life cycle [31,32]. *eIF4E-3*, *eIF4E-4*, *eIF4E-5*, and *eIF4E-7* transcripts are detected only from the third larval instar onwards, and *eIF4E-6* and *4E-HP* mRNAs are scarcely expressed in any stage of the life cycle [10,31]. Further studies showed mRNA expression in specific tissues [97,98]. A global picture of the functional diversification of *Drosophila* eIF4Es in different tissues is depicted in Figure 6.

**Figure 6.**
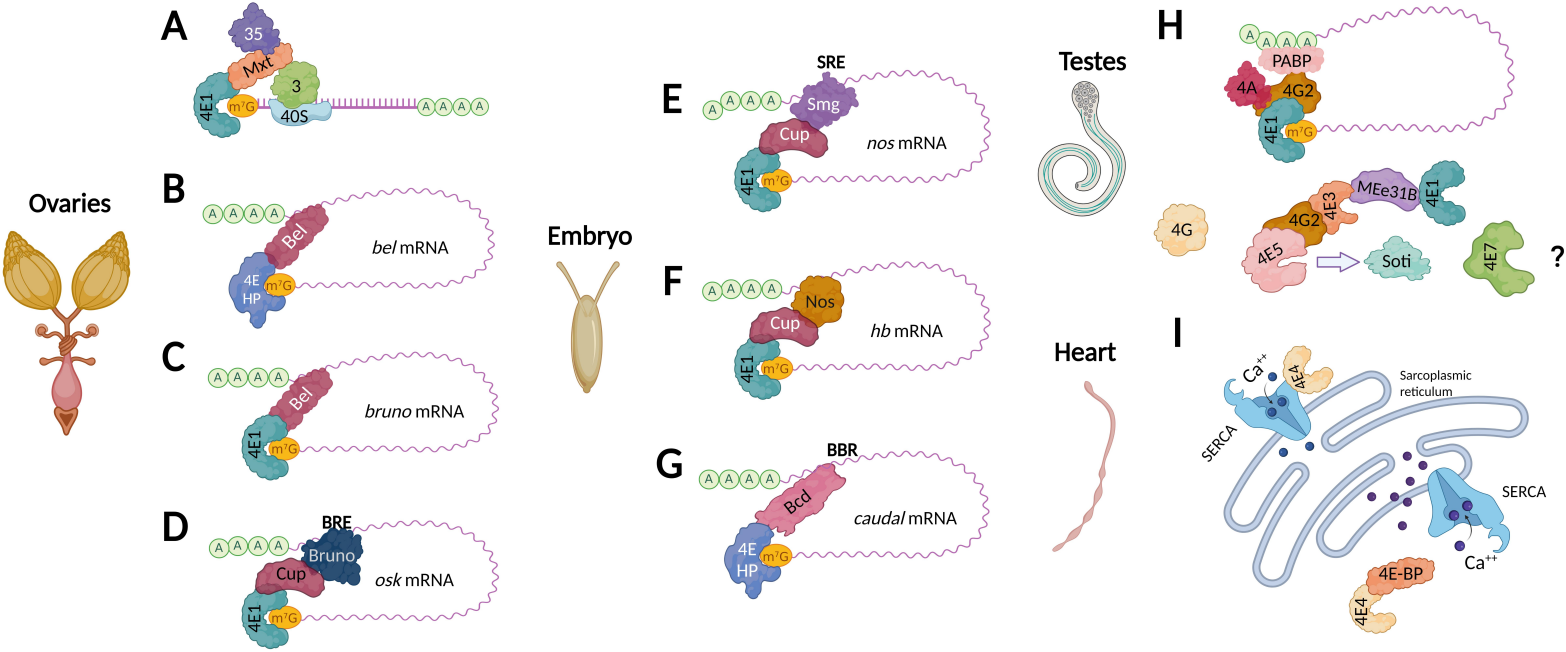
Roles of eIF4E isoforms in *D. melanogaster* development and heart tissue. *A-G)* eIF4E‑1 and 4E-HP bind different proteins to repress the translation of selected mRNAs during oogenesis *(A-D)* or embryogenesis *(E-G). A)* the modular protein Mextli binds to eIF4E‑1, the DEAD-box RNA helicase 35, and eIF3 to promote eIF4G-independent translation in ovaries. *B)* 4E-HP binds Belle to repress the translation of *belle* mRNA. *C)* eIF4E‑1 binds Belle to repress the translation of *bruno* mRNA. *D)* eIF4E‑1 binds cup in complex with Bruno to repress the translation of *oskar* mRNA. Bruno binds to the Bruno response element in the *oskar* 3´-UTR. *E)* eIF4E‑1 binds Cup in complex with smaug (*E*) or Nanos (*F*) to repress translation of *nanos* or *hunchback* mRNAs, respectively. *G)* 4E-HP binds Bicoid to repress translation of *caudal* mRNA. *H)* translation in adult testes. eIF4E‑1, eIF4E‑3, and eIF4E‑5 play crucial roles during spermatogenesis. The RNA helicase Me31B binds eIF4E‑1 and −3, possibly to repress translation. eIF4E‑5 is required for accumulation of soti at the distal ends of sperm tails, which promotes separation of mature sperm in a process called individualization. eIF4G, eIF4G2, and soti also play roles in the testis. *I)* Ca^++^ (*red dots*) uptake in adult cardiomyocytes is regulated by the calcium ATPase SERCA. Binding of eIF4E‑4 to SERCA decreases Ca^++^ uptake, whereas eIF4E‑4 sequestering by 4E-BP releases SERCA repression, thereby increasing Ca^++^ uptake into the sarcoplasmic reticulum. *4E-1*, eIF4E‑1; *4E-3*, eIF4E‑3; *4E‑5*, eIF4E‑5; *HP*, 4E-HP; *4 G*, eIF4G; *4G2*, eIF4G2; *Mxt*, Mextli; *35*, RNA helicase 35;* BRE*, Bruno response element; *bel, Belle; osk*, oskar; *Smg*, Smaug; *SRE*, smaug response element; *nos*, Nanos; *hb*, hunchback; *BBR*, Bicoid binding region;* Bcd*, Bicoid; *Pum*, pumilio; *X*, unidentified protein; *Me31B*, maternal expression at 31B; *4E-BP*, eIF4E-binding protein; *SERCA*, sarco-/endoplasmic reticulum Ca^2+^-ATPase; *S.R*., sarcoplasmic reticulum. Modified from [80]. Image created using BioRender.com. Elements representing testes were partially generated using Microsoft Copilot (powered by DALL-E) and edited by the authors to ensure scientific accuracy.

### Oogenesis and embryogenesis are regulated by eIF4E-1 and 4E-HP

During *Drosophila* oogenesis and embryogenesis, most maternal mRNAs are asymmetrically distributed and translationally repressed. At particular time -points and locations, their translation is activated by the action of various morphogens [96,99]. Coupling mRNA localization to translational control is key to achieving the right spatial and temporal distribution of proteins and to establishing body patterning [96]. Different metazoans utilize eIF4E-based translational repression to elicit correct spatiotemporal expression of specific proteins. In addition to their roles in routine translation and response to stresses, respectively, *Drosophila* eIF4E-1 and 4E-HP are crucially involved in these mechanisms by interacting with diverse proteins tethered to the 3´-UTR of selected mRNAs for translational repression [96]. 4E-HP cannot interact with eIF4G [10,11]. Thus, 4E-HP binding to the cap inhibits translation initiation of a subset of mRNAs.

Mextli (Mxt) is a scaffolding, eIF4G-like protein that, during oogenesis, binds directly to eIF4E-1 to form a cap-binding complex with eIF3 and the DEAD-box RNA helicase (DHX) 35 (encoded by *CG3225*) [19] (Figure 6(A)). There is no Mxt homolog in mammals. *mxt* plays a role in oogenesis and functions in germ line stem cell (GSC) maintenance during oogenesis. In early embryogenesis, embryos from *mxt* mutant females show a lower rate of hatching, severe defects in body segmentation, and other global defects [19]. The set of mRNAs whose translation is dependent upon Mxt remains unknown.

4E-HP binds the DEAD-box RNA helicase Belle (Bel). The complex 4E-HP–Bel is recruited to the *belle* (*bel*) mRNA cap, preventing Bel translation at the anterior oocyte border (Figure 6(B)). Subsequently, *bel* mRNA is transported to the posterior region of the oocyte, where the loss of 4E-HP repression alleviates translation of *bel* mRNA [21]. eIF4E-1 also binds Bel to inhibit Bruno mRNA translation at the posterior of the oocyte [21] (Figure 6(C)). When *bruno* translation is alleviated, Bruno interacts with Cup to recruit eIF4E-1 to *oskar* (*osk*) mRNA. Simultaneously, Bruno recognizes *osk* mRNA to prevent Oskar (Osk) expression [22,23] (Figure 6(D)).

During embryogenesis, eIF4E-1 binds Cup, which simultaneously interacts with Smaug tethered on *nanos* (*nos*) mRNA for translation repression at the anterior of the embryo [24] (Figure 6(E)). These interactions drive abdominal segmentation in the embryo. eIF4E-1 also binds Cup in complex with Nanos (Nos) on the 3´-UTR of *hunchback* (*hb*) mRNA to promote the formation of the abdominal and posterior embryonic structures [25] (Figure 6(F)). On the other hand, Bicoid (Bcd) binds the 3’-UTR of *caudal* (*cad*) mRNA. The simultaneous binding of 4E-HP to the cap of *cad* mRNA and Bcd blocks *cad* translation at the anterior, thereby generating an anterior-posterior protein gradient [29] (Figure 6(G)). At the posterior of the embryo, 4E-HP also forms a complex with brain tumour (Brat), Pumilio (Pum), and Nos to repress translation of *hb* mRNA to form an antero-posterior protein Hunchback (Hb) gradient along the longitudinal axis of the embryo. The opposing gradients of Caudal and Hunchback establish the head and thoracic segments of the fly [100–102].

### 4E-HP beyond embryogenesis

After embryogenesis, *4E-HP* also regulates synthesis of ecdysone, the major steroid hormone regulating molting and metamorphosis [103]. RNA interference (RNAi) experiments targeting *4E-HP* in the prothoracic gland disrupt ecdysone synthesis, blocking the transition from larva to pupa and affecting expression of steroidogenic enzymes. Moreover, *4E-HP*-null larvae show delayed development, fail to grow, and die [103]. The molecular mechanism by which 4E-HP regulates ecdysone synthesis remains unknown.

In cultured S2 cells, 4E-HP couples translational repression with decay of specific mRNAs. To do this, 4E-HP forms a complex with GIGYF to recruit the RNA helicase Me31B, the decapping activator HPat and the CCR4–NOT deadenylase complex as binding partners of GIGYF proteins [95]. However, the natural mRNA targets and functions of the 4E-HP–GIGYF complexes in different tissues and developmental stages are not known.

### eIF4E-1, eIF4E-3, and eIF4E-5 proteins are required for spermatogenesis

*Drosophila* spermatogenesis progresses when male germinal stem cells (GSC) divide asymmetrically to produce daughter cells called gonialblasts that undergo four mitotic amplification divisions to generate cysts of 16 spermatogonia, which then go through S-phase, at which point they become primary spermatocytes [104]. Primary spermatocytes undergo an extended G2 phase, followed by meitotic divisions to form cysts of 64 spermatids, which progress through cell elongation and individualization to give rise to mature sperm through a process called spermiogenesis [104]. Translational control is crucial for spermatogenesis. *eIF4E-3*, *eIF4E-4*, and *eIF4E-5* mRNAs are highly enriched in the testis [97,105]. eIF4E-3 and eIF4E-5 proteins are specifically expressed in the testis and, together with eIF4E-1, drive spermiogenesis. Indeed, eIF4E-1, eIF4E-3, and eIF4E-5 are essential for male fertility [12,28,106] (Figure 6(H)).

eIF4E-1 is expressed throughout spermatogenesis. Early expression in the germ cells and the surrounding somatic cyst cells is essential for gonad morphogenesis and germline development. Later, eIF4E-1 and eIF4E-3 act in a non-redundant manner to promote successful meiosis and spermatid differentiation [106]. eIF4E-3 is expressed in germline cells from the primary spermatocyte stage to the early differentiating spermatids before the onset of spermiogenesis, and is required for cytokinesis, meiotic chromosome segregation, and sperm individualization [12]. eIF4E-5 localizes to the cytoplasm of spermatocytes and to the distal ends of elongated spermatid cysts and is required for spermatid cyst polarization and individualization. eIF4E-5 regulates non-apoptotic caspase activity during individualization by promoting local accumulation of the E3 ubiquitin ligase inhibitor Soti at the distal ends of elongated spermatid cysts [28].

eIF4E-1 and eIF4E-3 interact with eIF4G and eIF4G-2 in testis to promote translation during spermatogenesis. eIF4G-2 is involved in meiotic progression and spermatid differentiation [12,107,108]. eIF4E-3 appears not to be regulated by 4E-BP in this tissue [12]. The RNA helicase Me31B is critical for spermatogenesis [109]. Me31B and eIF4E-3 colocalize in S2 cells, the testis stem cell niche, and spermatocytes. They also physically interact in yeast two-hybrid and FRET assays and coimmunoprecipitate from testis extracts, supporting the notion that Me31B is a negative regulator of translation in spermatogenesis by its association with eIF4E-3 [20].

eIF4E-7 possesses cap-binding activity [10,11]. Its mRNA is highly enriched in male reproductive organs and is also present in the fat body of pupae and third-instar larval imaginal disc of male individuals [97,98,110,111]. It binds eIF4G and eIF4G-2 *in vitro* (Table 1) and can rescue a yeast strain lacking endogenous eIF4E, showing that eIF4E-7 promotes translation [10]. The eIF4E-7 protein has been found in testes, forming a complex with Doublefault, a protein that controls mRNA translation in premeiotic spermatocytes [112], although a direct association with Doublefault was not demonstrated. Together, these findings suggest that eIF4E-7 promotes translation in spermatogenesis.

## eIF4E-4 and heart physiology

In *Drosophila*, heart performance relies on a tightly regulated handling of Ca^++^, a process in which the ATPase sarco-/endoplasmic reticulum Ca^++^-ATPase (SERCA) plays a pivotal role [113]. SERCA is regulated in the heart by 4E-BP during cardiac function [26]. Young flies over-expressing 4E-BP showed improved cardiac performance associated with increased SERCA activity [26]. Moreover, SERCA interacts with eIF4E-4 in the two-hybrid system, and eIF4E-4 mRNA and protein are present in the hearts of male adult flies [26]. These observations suggest that the SERCA and eIF4E‑4 might form a complex regulating the cardiac phenotype observed in 4E-BP over-expressing flies [26] (Figure 6(I)). Thus, eIF4E-4 might act as a negative regulator of SERCA during Ca^++^ uptake into the sarcoplasmic reticulum. This effect is alleviated when eIF4E-4 forms a complex with 4E-BP, allowing increased Ca^++^ uptake. Nonetheless, whether eIF4E-4 promotes translation in heart tissue remains unclear.

## The unexplored eIF4E-2 and eIF4E-6

The physiological roles of eIF4E-2 and eIF4E-6 are unknown. eIF4E-2 differs from eIF4E-1 only by a small stretch of amino acids at the N-terminus, whereas eIF4E-6 differs much more substantially at the N-terminus and C-terminus. eIF4E-2 and eIF4E-6 possess cap-binding activity [10,11,33], and bind to diverse eIF4E-interacting proteins *in vitro* (Table 1). However, neither eIF4E-2 nor eIF4E-6 was reported to form a functional complex *in vivo*. Their intracellular localization has not been addressed, nor is it known whether these proteins are regulated by the mTOR or MAPK cascades.

Despite the scarce information on eIF4E-2 and eIF4E-6, some findings on their biology have been reported. eIF4E-2 protein was isolated from post-ribosomal supernatant of total adult extracts and m^7^GTP-Sepharose column chromatography [33]. Although its mRNA is expressed throughout development [31], expression of the protein has not been explored during development or in specific tissues. It is able to bind eIF4G *in vitro* (Table 1) and can rescue a yeast strain lacking endogenous eIF4E [10], demonstrating that eIF4E-2 is a functional translation factor.

According to the large-scale analyses of mRNA profiles, modENCODERNA, Fly Cell Atlas, and FlyAtlas2 [97,98,110,111], eIF4E-6 mRNA appears to be male-specific and is enriched mostly in the testis, but is also present in third-instar larval imaginal discs, midgut, thoracicoabdominal ganglion, crop, brain, and head. eIF4E-6 does not rescue yeast lacking endogenous eIF4E [10] nor does it bind eIF4G *in vitro* [10,11]. This suggests eIF4E-6 might be a repressor of translation of specific mRNAs in males. However, no experiments showing protein expression in specific tissues or identifying potential binding partners have been published. Moreover, phenotypes of *eIF4E-6* mutants have not yet been described.

## Outlook

eIF4E has emerged as a protein that associates with diverse partners to perform distinct roles in gene expression and mRNA metabolism. *Drosophila* paralogs eIF4E-1, −3, −4, −5, and 4E-HP are key players in different processes, hand in hand with their different interactors. For eIF4E-2, −6, and 7, no specific function has yet been reported, although eIF4E-2 and 7 promote translation. Unveiling their actual partners *in vivo* will be key to discovering their specific roles in *Drosophila* biology. A clue to their possible functions is suggested by their expression patterns in the different tissues, but the actual presence of the proteins should be corroborated using specific antibodies and Western blotting, m^7^GTP-Sepharose column chromatography, or proteomic approaches. The study of existing mutant alleles also should be instrumental in unveiling their *in vivo* roles.

eIF4E-1, eIF4E-3, and eIF4E-5 play roles during spermatogenesis, and the data reported suggest eIF4E-6 and −7 might also play roles in the testis. However, it is unclear why the male, but not the female, gonad, requires several eIF4Es. On the other hand, eIF4E-1 plays a role in splicing of the *Sex-lethal* (*Sxl*) pre-mRNA during female sex determination, acting as a co-factor in the female-specific splicing of *Msl-2* and *Sxl* mRNAs [77]. Thus, it appears that the emergence of several eIF4E paralogs correlates with the evolution of several sex-specific features and processes in *Drosophila*.

Addressing the functions of all *Drosophila* eIF4Es will shed light on the functional divergence of these proteins in RNA metabolism, gene expression, and development.

## Data Availability

Data sharing is not applicable. All new data for this Review are depicted in Figures 1 and 2, and Table 2. No other data have been generated or analyzed.

## References

[1] Sonenberg N. eIF4E, the mRNA cap-binding protein: from basic discovery to translational researchThis paper is one of a selection of papers published in this special issue, entitled CSBMCB — systems and chemical biology, and has undergone the Journal’s usual peer review process. Biochem Cell Biol. 2008;86(2):178–183. doi: 10.1139/O08-034 18443631

[2] Pelletier J, Sonenberg N. The organizing principles of eukaryotic ribosome recruitment. Annu Rev Biochem. 2019;88(1):307–335. doi: 10.1146/annurev-biochem-013118-111042 31220979

[3] Hernández G. The versatile relationships between eIF4E and eIF4E-interacting proteins. Trends Genet. 2022;38(8):801–804. doi: 10.1016/j.tig.2022.04.003 35568601

[4] Tettweiler G, Kowanda M, Lasko P, et al. The distribution of eIF4E-family members across Insecta. Comp Funct Genom. 2012;2012:1–15. doi: 10.1155/2012/960420 PMC3382400 22745595

[5] Zhimulev IF, Koryakov DE. Polytene chromosomes. Encyclopedia of Life Sciences. Chichester: John Wiley & Sons; 2009.

[6] Painter TS. Salivary chromosomes and the attack on the gene. J Heredity. 1934;25(12):465–476. doi: 10.1093/oxfordjournals.jhered.a103862

[7] Hernández G, Ross-Kaschitza D, Moreno-Hagelsieb G, et al. Analysis of eIF4E-family members in fungi contributes to their classification in eukaryotes. J Biol Chem. 2025;301(2):108129. doi: 10.1016/j.jbc.2024.108129 PMC11791286 39716494

[8] Rhoads RE. eIF4E: new family members, new binding partners, new roles. J Biol Chem. 2009;284(25):16711–16715. doi: 10.1074/jbc.R900002200 PMC2719305 19237539

[9] Zapata JM, Martínez MA, Sierra JM. Purification and characterization of eukaryotic polypeptide chain initiation factor 4F from *Drosophila melanogaster* embryos. J Biol Chem. 1994;269(27):18047–18052. doi: 10.1016/S0021-9258(17)32415-8 8027064

[10] Hernández G, Altmann M, Sierra JM, et al. Functional analysis of seven genes encoding eight translation initiation factor 4E (eIF4E) isoforms in *Drosophila*. Mech Dev. 2005;122(4):529–543. doi: 10.1016/j.mod.2004.11.011 15804566

[11] Zuberek J, Kuchta K, Hernández G, et al. Diverse cap-binding properties of *drosophila* eIF4E isoforms. Biochim Biophys Acta. 2016;1864(10):1292–1303. doi: 10.1016/j.bbapap.2016.06.015 27374989

[12] Hernández G, Han H, Gandin V, et al. Eukaryotic initiation factor 4E-3 is essential for meiotic chromosome segregation, cytokinesis and male fertility in *Drosophila*. Development. 2012;139(17):3211–3220. doi: 10.1242/dev.073122 PMC3413165 22833128

[13] Igreja C, Peter D, Weiler C, et al. 4E-BPs require non-canonical 4E-binding motifs and a lateral surface of eIF4E to repress translation. Nat Commun. 2014;5(1):4790. doi: 10.1038/ncomms5790 PMC4164784 25179781

[14] Kinkelin K, Veith K, Grunwald M, et al. Crystal structure of a minimal eIf4E-Cup complex reveals a general mechanism of eIF4E regulation in translational repression. RNA. 2012;18(9):1624–1634. doi: 10.1261/rna.033639.112 PMC3425778 22832024

[15] Peter D, Igreja C, Weber R, et al. Molecular architecture of 4E-BP translational inhibitors bound to eIF4E. Mol Cell. 2015;57(6):1074–1087. doi: 10.1016/j.molcel.2015.01.017 25702871

[16] Peter D, Weber R, Kone C, et al. Mextli proteins use both canonical bipartite and novel tripartite binding modes to form eIF4E complexes that display differential sensitivity to 4E-BP regulation. Genes Dev. 2015;29(17):1835–1849. doi: 10.1101/gad.269068.115 PMC4573856 26294658

[17] Gruner S, Peter D, Weber R, et al. The structures of eIF4E-eIF4G complexes reveal an extended interface to regulate translation initiation. Mol Cell. 2016;64(3):467–479. doi: 10.1016/j.molcel.2016.09.020 27773676

[18] Hernández G, Castellano MM, Agudo M, et al. Isolation and characterization of the cDNA and the gene for eukaryotic translation initiation factor 4G from *Drosophila melanogaster*. Eur J Biochem. 1998;253(1):27–35. doi: 10.1046/j.1432-1327.1998.2530027.x 9578457

[19] Hernández G, Miron M, Han H, et al. Mextli is a novel eukaryotic translation initiation factor 4E-binding protein that promotes translation in *Drosophila melanogaster*. Mol Cell Biol. 2013;33(15):2854–2864. doi: 10.1128/MCB.01354-12 PMC3719689 23716590

[20] Layana C, Vilardo ES, Corujo G, et al. *Drosophila* Me31B is a dual eIF4E-interacting protein. J Mol Biol. 2023;435(5):167949. doi: 10.1016/j.jmb.2023.167949 36638908

[21] Yarunin A, Harris RE, Ashe MP, et al. Patterning of the *drosophila* oocyte by a sequential translation repression program involving the d4EHP and Belle translational repressors. RNA Biol. 2011;8(5):904–912. doi: 10.4161/rna.8.5.16325 21788736

[22] Nakamura A, Sato K, Hanyu-Nakamura K. *Drosophila* cup is an eIF4E binding protein that associates with Bruno and regulates *oskar* mRNA translation in oogenesis. Dev Cell. 2004;6(1):69–78. doi: 10.1016/S1534-5807(03)00400-3 14723848

[23] Wilhelm JE, Hilton M, Amos Q, et al. Cup is an eIF4E binding protein required for both the translational repression of oskar and the recruitment of Barentsz. J Cell Biol. 2003;163(6):1197–1204. doi: 10.1083/jcb.200309088 PMC2173729 14691132

[24] Nelson MR, Leidal AM, Smibert CA. *Drosophila* cup is an eIF4E-binding protein that functions in Smaug-mediated translational repression. EMBO J. 2004;23(1):150–159. doi: 10.1038/sj.emboj.7600026 PMC1271664 14685270

[25] Zappavigna V, Piccioni F, Villaescusa JC, et al. Cup is a nucleocytoplasmic shuttling protein that interacts with the eukaryotic translation initiation factor 4E to modulate *drosophila* ovary development. Proc Natl Acad Sci USA. 2004;101(41):14800–14805. doi: 10.1073/pnas.0406451101 PMC522052 15465908

[26] Santalla M, García A, Mattiazzi A, et al. Interplay between SERCA, 4E-BP, and eIF4E in the *drosophila* heart. PLOS ONE. 2022;17(5):e0267156. doi: 10.1371/journal.pone.0267156 PMC9119464 35588119

[27] Niessing D, Blanke S, Jäckle H. Bicoid associates with the 5’-cap-bound complex of caudal mRNA and represses translation. Genes Dev. 2002;16(19):2576–2582. 10.1101/gad.240002 PMC187448 12368268

[28] Shao L, Fingerhut JM, Falk BL, et al. Eukaryotic translation initiation factor eIf4E-5 is required for spermiogenesis in *Drosophila melanogaster*. Development. 2023;150(4):dev200477. doi: 10.1242/dev.200477 36695474

[29] Cho PF, Poulin F, Cho-Park YA, et al. A new paradigm for translational control: inhibition via 5’-3’ mRNA tethering by Bicoid and the eIF4E cognate 4E-HP. Cell. 2005;121(3):411–423. doi: 10.1016/j.cell.2005.02.024 15882623

[30] Maroto FG, Sierra JM. Purification and characterization of mRNA cap-binding protein from *Drosophila melanogaster* embryos. Mol Cell Biol. 1989;9(5):2181–2190. doi: 10.1128/mcb.9.5.2181-2190.1989 PMC363012 2501660

[31] Hernández G, Sierra JM. Translation initiation factor eIF-4E from *Drosophila*: cDNA sequence and expression of the gene. Biochim Biophys Acta. 1995;1261(3):427–431. doi: 10.1016/0167-4781(95)00039-J 7742371

[32] Hernández G, Diez Del Corral R, Santoyo J, et al. Localization, structure and expression of the gene for translation initiation factor eIF4E from *Drosophila melanogaster*. Mol Gen Genet. 1997;253(5):624–633. doi: 10.1007/s004380050365 9065696

[33] Lavoie CA, Lachance P, Sonenberg N, et al. Alternatively spliced transcripts from the *drosophila* eIF4E gene produce two different cap-binding proteins. J Biol Chem. 1996;271(27):16393–16398. 10.1074/jbc.271.27.16393 8663200

[34] Reyes R, Izquierdo JM. Half pint couples transcription and splicing of *eIf4E-1,2* gene during fly development. Biochem Biophys Res Comm. 2008;374(4):758–762. doi: 10.1016/j.bbrc.2008.07.104 18671938

[35] Van Buskirk C, Schupbach T. *Half pint* regulates alternative splice site selection in *Drosophila*. Dev Cell. 2002;2(3):343–353. doi: 10.1016/S1534-5807(02)00128-4 11879639

[36] Lasko P. The *Drosophila melanogaster* genome: translational factors and RNA binding proteins. J Cell Biol. 2000;150(2):F51–F6. doi: 10.1083/jcb.150.2.F51 PMC2180218 10908586

[37] Roux PP, Topisirovic I. Signaling pathways involved in the regulation of mRNA translation. Mol Cell Biol. 2018;38(12):e00070–18. doi: 10.1128/MCB.00070-18 PMC5974435 29610153

[38] Proud CG. Phosphorylation and signal transduction pathways in translational control cold Spring. Cold Spring Harb Perspect Biol. 2019;11(7):a033050. doi: 10.1101/cshperspect.a033050 PMC6601458 29959191

[39] Gingras AC, Raught B, Sonenberg N. eIF4 initiation factors: effectors of mRNA recruitment to ribosomes and regulators of translation. Annu Rev Biochem. 1999;68(1):913–963. doi: 10.1146/annurev.biochem.68.1.913 10872469

[40] Bernal A, Kimbrell DA. *Drosophila* Thor participates in host immune defense and connects a translational regulator with innate immunity. Proc Natl Acad Sci USA. 2000;97(11):6019–6024. doi: 10.1073/pnas.100391597 PMC18551 10811906

[41] Flatt T, Min KJ, D’alterio C, et al. *Drosophila* germ-line modulation of insulin signaling and lifespan. Proc Natl Acad Sci USA. 2008;105(17):6368–6373. doi: 10.1073/pnas.0709128105 PMC2359818 18434551

[42] Miron M, Lasko P, Sonenberg N. Signaling from Akt to FRAP/TOR targets both 4E-BP and S6K in *Drosophila melanogaster*. Mol Cell Biol. 2003;23(24):9117–9126. doi: 10.1128/MCB.23.24.9117-9126.2003 PMC309682 14645523

[43] Miron M, Verdu J, Lachance PE, et al. The translational inhibitor 4E-BP is an effector of PI(3)K/Akt signaling and cell growth. Nat Cell Biol. 2001;3(6):596–601. doi: 10.1038/35078571 11389445

[44] Teleman AA, Chen YW, Cohen SM. 4E-BP functions as a metabolic brake used under stress conditions but not during normal growth. Genes Dev. 2005;19(16):1844–1848. doi: 10.1101/gad.341505 PMC1186183 16103212

[45] Lachance PE, Miron M, Raught B, et al. Phosphorylation of eukaryotic translation initiation factor 4E is critical for growth. Mol Cell Biol. 2002;22(6):1656–16663. doi: 10.1128/MCB.22.6.1656-1663.2002 PMC135594 11865045

[46] Yang H, Zingaro VA, Lincoff J, et al. Remodelling of the translatome controls diet and its impact on tumorigenesis. Nature. 2024;633(8028):189–197. doi: 10.1038/s41586-024-07781-7 PMC12147720 39143206

[47] Ruan H, Li X, Xu X, et al. eIF4E S209 phosphorylation licenses myc- and stress-driven oncogenesis. Elife. 2020;9:e60151. doi: 10.7554/eLife.60151 PMC7665890 33135632

[48] Furic L, Rong W, Larsson O, et al. eIF4E phosphorylation promotes tumorigenesis and is associated with prostate cancer progression. Proc Natl Acad Sci USA. 2010;107(32):14134–14139. 10.1073/pnas.1005320107 PMC2922605 20679199

[49] Arquier N, Bourouis M, Colombani J, et al. *Drosophila* Lk6 kinase controls phosphorylation of eukaryotic translation initiation factor 4E and promotes normal growth and development. Curr Biol. 2005;15(1):19–23. 10.1016/j.cub.2004.12.037 15649359

[50] Parra-Palau JL, Scheper GC, Harper DE, et al. The *drosophila* protein kinase LK6 is regulated by erk and phosphorylates the eukaryotic initiation factor eIF4E *in vivo*. Biochem J. 2005;385(Pt 3):695–702. 10.1042/BJ20040769 PMC1134744 15487973

[51] Reiling JH, Doepfner KT, Hafen E, et al. Diet-dependent effects of the *drosophila* Mnk1/Mnk2 homolog Lk6 on growth via eIF4E. Curr Biol. 2005;15(1):24–30. doi: 10.1016/j.cub.2004.12.034 15649360

[52] Marcotrigiano J, Gingras AC, Sonenberg N, et al. Cocrystal structure of the messenger RNA 5’ cap-binding protein (eIF4E) bound to 7-methyl-GDP. Cell. 1997;89(6):951–961. doi: 10.1016/S0092-8674(00)80280-9 9200613

[53] Matsuo H, Li H, McGuire AM, et al. Structure of translation factor eIF4E bound to m7GDP and interaction with 4E-binding protein. Nat Struct Biol. 1997;4(9):717–724. 10.1038/nsb0997-717 9302999

[54] Tomoo K, Shen X, Okabe K, et al. Crystal structures of 7-methylguanosine 5’-triphosphate (m(7)GTP)- and P(1)-7-methylguanosine-P(3)-adenosine-5’,5’-triphosphate (m(7)GpppA)-bound human full-length eukaryotic initiation factor 4E: biological importance of the C-terminal flexible region. Biochem J. 2002;362(3):539–544. doi: 10.1042/bj3620539 PMC1222416 11879179

[55] Tomoo K, Shen X, Okabe K, et al. Structural features of human initiation factor 4E, studied by X-ray crystal analyses and molecular dynamics simulations. J Mol Biol. 2003;328(2):365–383. doi: 10.1016/S0022-2836(03)00314-0 12691746

[56] Mader S, Lee H, Pause A, et al. The translation initiation factor eIF-4E binds to a common motif shared by the translation factor eIF-4 gammaand the translational repressors 4E-binding proteins. Mol Cell Biol. 1995;15(9):4990–4997. doi: 10.1128/MCB.15.9.4990 PMC230746 7651417

[57] Haghighat A, Mader S, Pause A, et al. Repression of cap-dependent translation by 4E-binding protein 1: competition with p220 for binding to eukaryotic initiation factor-4E. EMBO J. 1995;14(22):5701–5709. doi: 10.1002/j.1460-2075.1995.tb00257.x PMC394685 8521827

[58] Hernández G, Gillespie KM, Bachvaroff TS, et al. Evolution of eIf4E-Interacing proteins. In: Hernández G Jagus R, editors. Evolution of the protein synthesis machinery and its regulation. Switzerland: Springer; 2016. p. 207–234.

[59] Grüner S, Weber R, Peter D, et al. Structural motifs in eIF4G and 4E-BPs modulate their binding to eIF4E to regulate translation initiation in yeast. Nucleic Acid Res. 2018;46(13):6893–6908. doi: 10.1093/nar/gky542 PMC6061780 30053226

[60] Gross JD, Moerke NJ, von der Haar T, et al. Ribosome loading onto the mRNA cap is driven by conformational coupling between eIF4G and eIF4E. Cell. 2003;115(6):739–750. doi: 10.1016/S0092-8674(03)00975-9 14675538

[61] Jumper J, Evans R, Pritzel A, et al. Highly accurate protein structure prediction with AlphaFold. Nature. 2021;596(7873):583–589. doi: 10.1038/s41586-021-03819-2 PMC8371605 34265844

[62] Freire ER, Sturm NR, Campbell DA, et al. The role of cytoplasmic mRNA cap-binding protein complexes in *Trypanosoma brucei* and other trypanosomatids. Pathogens. 2017;6:55. doi: 10.3390/pathogens6040055 PMC5750579 29077018

[63] Hernández G, Lalioti V, Vanderkerckhove J, et al. Identification and characterization of the expression of the translation initiation factor 4A (eIF4A) from *Drosophila melanogaster*. Proteomics. 2004;4(2):316–326. doi: 10.1002/pmic.200300555 14760701

[64] Maroto FG, Sierra JM. Translational control in heat-shocked drosophila embryos. Evidence for the inactivation of initiation factor(s) involved in the recognition of mRNA cap structure. J Biol Chem. 1988;263(30):15720–15725. doi: 10.1016/S0021-9258(19)37647-1 3139675

[65] Hernández G, Vazquez-Pianzola P, Sierra JM, et al. Internal ribosome entry site drives cap-independent translation of reaper and heat shock protein 70 mRNAs in *drosophila* embryos. RNA. 2004;10(11):1783–1797. doi: 10.1261/rna.7154104 PMC1370666 15496524

[66] McNeill H, Craig GM, Bateman JM. Regulation of neurogenesis and epidermal growth factor receptor signaling by the insulin receptor/target of rapamycin pathway in *Drosophila*. Genetics. 2008;179(2):843–853. doi: 10.1534/genetics.107.083097 PMC2429878 18505882

[67] Liang M, Hody C, Yammine V, et al. eIF4EHP promotes ldh mRNA translation in and fruit fly adaptation to hypoxia. EMBO Rep. 2023;24(7):e56460. doi: 10.15252/embr.202256460 PMC10328074 37144276

[68] Hipp MS, Kasturi P, Hartl FU. The proteostasis network and its decline in ageing. Nat Rev Mol Cell Biol. 2019;20(7):421–435. doi: 10.1038/s41580-019-0101-y 30733602

[69] Vattem KM, Wek RC. Reinitiation involving upstream ORFs regulates ATF4 mRNA translation in mammalian cells. Proc Natl Acad Sci USA. 2004;101(31):11269–11274. doi: 10.1073/pnas.0400541101 PMC509193 15277680

[70] Walsh K, Katow H, Junn H, et al. 4EHP and NELF-E regulate physiological ATF4 induction and proteostasis in disease models of *drosophila*. Nat Comm. 2025;17(1):626. doi: 10.1038/s41467-025-67357-5 PMC12816580 41436469

[71] Topisirovic I, Kentsis A, Perez JM, et al. Eukaryotic translation initiation factor 4E activity is modulated by HOXA9 at multiple levels. Mol Cell Biol. 2005;25(3):1100–1112. doi: 10.1128/MCB.25.3.1100-1112.2005 PMC544005 15657436

[72] Lejbkowicz F, Goyer C, Darveau A, et al. A fraction of the mRNA 5’ cap-binding protein, eukaryotic initiation factor 4E, localizes to the nucleus. Proc Natl Acad Sci USA. 1992;89(20):9612–9616. doi: 10.1073/pnas.89.20.9612 PMC50182 1384058

[73] Lai HK, Borden KL. The promyelocytic leukemia (PML) protein suppresses cyclin D1 protein production by altering the nuclear cytoplasmic distribution of cyclin D1 mRNA. Oncogene. 2000;19(13):1623–1634. doi: 10.1038/sj.onc.1203473 10763819

[74] Rosenwald IB, Kaspar R, Rousseau D, et al. Eukaryotic translation initiation factor 4E regulates expression of cyclin D1 at transcriptional and post-transcriptional levels. J Biol Chem. 1995;270(36):21176–21180. doi: 10.1074/jbc.270.36.21176 7673150

[75] Rousseau D, Kaspar R, Rosenwald I, et al. Translation initiation of ornithine decarboxylase and nucleocytoplasmic transport of cyclin D1 mRNA are increased in cells overexpressing eukaryotic initiation factor 4E. Proc Natl Acad Sci USA. 1996;93(3):1065–1070. doi: 10.1073/pnas.93.3.1065 PMC40031 8577715

[76] Rong L, Livingstone M, Sukarieh R, et al. Control of eIF4E cellular localization by eIF4E-binding proteins, 4E-BPs. RNA. 2008;14(7):1318–1327. doi: 10.1261/rna.950608 PMC2441981 18515545

[77] Graham PL, Yanowitz JL, Penn JKM, et al. The translation initiation factor eIF4E regulates the sex-specific expression of the master switch gene *sxl* in *Drosophila melanogaster*. PLoS Genet. 2011;7(7):e1002185. doi: 10.1371/journal.pgen.1002185 PMC3145617 21829374

[78] Davis MR, Delaleau M, Borden KLB. Nuclear eIF4E stimulates 3´-end cleavage of target RNAs. Cell Rep. 2019;27(5):1397–1408. doi: 10.1016/j.celrep.2019.04.008 PMC6661904 31042468

[79] Osborne MJ, Borden KLB. The eukaryotic translation initiation factor eIF4E in the nucleus: taking the road less traveled. Immunol Rev. 2014;263(1):210–223. doi: 10.1111/imr.12240 PMC4269245 25510279

[80] Hernández G, Vazquez-Pianzola P. eIF4E as a molecular wildcard in metazoans RNA metabolism. Biol Rev Cambr Phil Soc. 2023;98(6):2284–2306. doi: 10.1111/brv.13005 37553111

[81] Freire ER, Dhalia R, Moura DM, et al. The four trypanosomatid eIF4E homologues fall into two separate groups, with distinct features in primary sequence and biological properties. Mol Biochem Parasitol. 2011;176(1):25–36. doi: 10.1016/j.molbiopara.2010.11.011 PMC6736675 21111007

[82] Li L, Wang CC. Identification in the ancient protist *Giardia lamblia* of two eukaryotic translation initiation factor 4E homologues with distinctive functions. Eukaryot Cell. 2005;4:948–959. doi: 10.1128/EC.4.5.948-959.2005 PMC1140097 15879529

[83] Strudwick S, Borden KL. The emerging roles of translation factor eIF4E in the nucleus. Differentiation. 2002;70(1):10–22. doi: 10.1046/j.1432-0436.2002.700102.x 11963652

[84] Lang V, Zanchin NIT, Lunsdorf H, et al. Initiation factor eIF-4E of *Saccharomyces cerevisiae*. distribution within the cell, binding to mRNA, and consequences of its overproduction. J Biol Chem. 1994;269(8):6117–6123. doi: 10.1016/S0021-9258(17)37577-4 8119957

[85] Vélez DE, Ristic Z, Ross-Kaschitza D, et al. Med9 is a novel eIF4E-interacting protein in *Saccharomyces cerevisiae*. Int J Mol Sci. 2026;27(15):6911. 10.3390/ijms27156911 PMC13467561 42589564

[86] Cohen N, Sharma M, Kentsis A, et al. PML RING suppresses oncogenic transformation by reducing the affinity of eIF4E for mRNA. EMBO J. 2001;20(16):4547–4559. doi: 10.1093/emboj/20.16.4547 PMC125576 11500381

[87] Youn JY, Dyakov BJA, Zhang J, et al. Properties of stress granule and P-body proteomes. Mol Cell. 2019;76(2):286–294. doi: 10.1016/j.molcel.2019.09.014 31626750

[88] Buchan JR, Parker R. Eukaryotic stress granules: the ins and outs of translation. Mol Cell. 2009;36(6):932–941. doi: 10.1016/j.molcel.2009.11.020 PMC2813218 20064460

[89] Eulalio A, Behm-Ansmant I, Schweizer D, et al. P-body formation is a consequence, not the cause, of RNA-mediated gene silencing. Mol Cell Biol. 2006;27(11):3970–3981. doi: 10.1128/MCB.00128-07 PMC1900022 17403906

[90] Thomson T, Liu N, Arkov A, et al. Isolation of new polar granule components in *Drosophila* reveals P body and er associated proteins. Mech Dev. 2008;125(9–10):865–873. doi: 10.1016/j.mod.2008.06.005 PMC2570953 18590813

[91] DeHaan H, McCambridge A, Armstrong B, et al. An *in vivo* proteomic analysis of the Me31B interactome in *drosophila* germ granules. FEBS Lett. 2017;591(21):3536–3547. doi: 10.1002/1873-3468.12854 28945271

[92] McCambridge A, Solanki D, Olchawa N, et al. Comparative proteomics reveal Me31B’s interactome dynamics, expression regulation, and assembly mechanism into germ granules during *drosophila* germline development. Sci Rep. 2020;10(1):564. doi: 10.1038/s41598-020-57492-y PMC6969142 31953495

[93] Wang M, Ly M, Lugowski A, et al. ME31B globally represses maternal mRNAs by two distinct mechanisms during the *drosophila* maternal-to-zygotic transition. Elife. 2017;6:e27891. doi: 10.7554/eLife.27891 PMC5779226 28875934

[94] Ferrero PV, Layana C, Paulucci E, et al. Cap binding-independent recruitment of eIF4E to cytoplasmic foci. Biochim Biophys Acta. 2012;1823(7):1217–1224. doi: 10.1016/j.bbamcr.2012.03.013 22507384

[95] Ruscica V, Bawankar P, Peter D, et al. Direct role for the *drosophila* GIGYF protein in 4EHP-mediated mRNA repression. Nucleic Acid Res. 2019;47(13):7035–7048. doi: 10.1093/nar/gkz429 PMC6648886 31114929

[96] Kong J, Lasko P. Translational control in cellular and developmental processes. Nat Rev Genet. 2012;13(6):383–394. doi: 10.1038/nrg3184 22568971

[97] Graveley BR, Brooks AN, Carlson JW, et al. The developmental transcriptome of *Drosophila melanogaster*. Nature. 2011;471(7339):473–479. doi: 10.1038/nature09715 PMC3075879 21179090

[98] Brown JB, Boley N, Eisman R, et al. Diversity and dynamics of the *drosophila* transcriptome. Nature. 2014;512(7515):393–399. doi: 10.1038/nature12962 PMC4152413 24670639

[99] Lécuyer E, Yoshida H, Krause HM. Global implications of mRNA localization pathways in cellular organization. Curr Opin Cell Biol. 2009;21(3):409–415. doi: 10.1016/j.ceb.2009.01.027 19249199

[100] Cho PF, Gamberi C, Cho-Park YA, et al. Cap-dependent translational inhibition establishes two opposing morphogen gradients in *drosophila* embryos. Curr Biol. 2006;16(20):2031–2041. doi: 10.1016/j.cub.2006.08.093 PMC2238800 17055983

[101] Sonoda J, Wharton RP. Recruitment of Nanos to *hunchback* mRNA by Pumilio. Genes Dev. 1999;13(20):2704–2712. doi: 10.1101/gad.13.20.2704 PMC317116 10541556

[102] Sonoda J, Wharton RP. *Drosophila* brain tumor is a translational repressor. Genes Dev. 2001;15(6):762–773. doi: 10.1101/gad.870801 PMC312658 11274060

[103] Valzania L, Ono H, Ignesti M, et al. *Drosophila* 4EHP is essential for the larval–pupal transition and required in the prothoracic gland for ecdysone biosynthesis. Dev Biol. 2016;410(1):14–23. doi: 10.1016/j.ydbio.2015.12.021 26721418

[104] Fabian L, Brill JA. *Drosophila* spermiogenesis: big things come from little packages. Spermatogenesis. 2012;2(3):197–212. doi: 10.4161/spmg.21798 PMC3469442 23087837

[105] Krause SA, Overend G, Dow JAT, et al. FlyAtlas 2 in 2022: enhancements to the *Drosophila melanogaster* expression atlas. Nucleic Acid Res. 2022;50(1):D1010–D5. 10.1093/nar/gkab971 PMC8728208 34718735

[106] Ghosh S, Lasko P. Loss-of-function analysis reveals distinct requirements of the translation initiation factors eIF4E, eIf4E-3, eIF4G and eIF4G2 in *drosophila* spermatogenesis. PLOS ONE. 2015;10(4):e0122519. doi: 10.1371/journal.pone.0122519 PMC4388691 25849588

[107] Baker CC, Fuller MT. Translational control of meiotic cell cycle progression and spermatid differentiation in male germ cells by a novel eIF4G homolog. Development. 2007;134(15):2863–2869. doi: 10.1242/dev.003764 PMC4620998 17611220

[108] Franklin-Dumont M, Chatterjee C, Wasserman SA, et al. A novel eIF4G homolog, off-schedule, couples translational control of meiosis and differentiation in *drosophila* spermatocytes. Development. 2007;134(15):2851–2861. doi: 10.1242/dev.003517 17611222

[109] Jensen L, Venkei ZG, Watase GJ, et al. Me31B regulates stem cell homeostasis by preventing excess dedifferentiation in the *drosophila* male germline. J Cell Sci. 2021;134(14):jcs258757. doi: 10.1242/jcs.258757 PMC8325955 34164657

[110] Chintapalli VR, Wang J, Dow JAT. Using FlyAtlas to identify better *Drosophila melanogaster* models of human disease. Nat Gen. 2007;39(6):715–720. doi: 10.1038/ng2049 17534367

[111] Li H, Janssens J, De Waegeneer M, et al. Fly cell atlas: a single-nucleus transcriptomic atlas of the adult fruit fly. Science. 2022;375(6584):eabk2432. doi: 10.1126/science.abk2432 PMC8944923 35239393

[112] Sechi S, Frappaolo A, Karimpour-Ghahnavieh A, et al. *Drosophila* doublefault protein coordinates multiple events during male meiosis by controlling mRNA translation. Development. 2019;146:dev183053. doi: 10.1242/dev.183053 PMC6899030 31645358

[113] Gambardella J, Trimarco B, Laccarino G, et al. New insights in cardiac calcium handling and excitation-contraction coupling. Adv Exp Med Biol. 2018;1067:373–385 doi: 10.1007/5584_2017_106 PMC5889357 28956314

